# Abnormal cisatracurium pharmacodynamics and pharmacokinetics among patients with severe aortic regurgitation during anesthetic induction

**DOI:** 10.1186/s12871-020-0935-z

**Published:** 2020-01-22

**Authors:** Xiaocong Huang, Lei Chen, Yujing Cai, Jinfeng Wei, Lina Lin, Jie Sun, Xuemei Peng, Sheng Wang

**Affiliations:** 1 Department of Anesthesiology, Guangdong Provincial Cardiovascular Institute, Guangdong Provincial People’s Hospital (previously called Guangdong General Hospital), Guangdong Academy of Medical Sciences, Guangzhou, 510630 China; 2 Department of Anesthesiology, the First Affiliated Hospital of Jinan University, Guangzhou, 510632 China; 3 Department of Anesthesiology, Guangdong Provincial People’s Hospital (previously called Guangdong General Hospital), Guangdong Academy of Medical Sciences, Guangzhou, 510630 China; 4 Department of Anesthesiology, Guangdong Provincial People’s Hospital (previously called Guangdong General Hospital), Guangzhou, 510630 China; 5 College of Pharmacy, Jinan University, Guangzhou, 510632 China; 6 Department of Anesthesiology, Linzhi People’s Hospital, Xizang, 860000 China

**Keywords:** Cisatracurium, Severe aortic regurgitation, Neuromuscular blockade, Intercompartment transfer rate

## Abstract

**Background:**

This study was designed to examine whether severe aortic regurgitation will affect the pharmacodynamics (PD) and pharmacokinetics (PK) of cisatracurium during anesthetic induction.

**Methods:**

A total of 32 patients were divided into two groups: the AR group (*n* = 16) and the control group (n = 16). Arterial blood samples were drawn before and at 1, 2, 4, 6, 8, 10, 16 and 20 min after intravenous injection of 0.15 mg/kg cisatracurium. TOF tests were applied to determine the onset time of maximal muscle relaxation. The concentration of cisatracurium in plasma was determined by high-performance liquid chromatography.

**Results:**

The onset time to maximal neuromuscular block was prolonged from 2.07 ± 0.08 min to 4.03 ± 0.14 min, which indicated that the PD responses to cisatracurium were significantly delayed in the AR group (*P* < 0.05) compared to the control group.

A conventional two-compartment PK model showed a higher plasma concentration of cisatracurium among the AR group with markedly reduced intercompartment transfer rate (K_12_ = 0.19 ± 0.02 and K_21_ = 0.11 ± 0.01 in the AR group vs. K_12=_0.26 ± 0.01 and K_21_ = 0.19 ± 0.01 in the control group, *P* < 0.01) compared to the control group.

**Conclusion:**

Backward blood flow during diastole in severe AR impaired distribution of cisatracurium from the central compartment to the peripheral compartment, which accounted for the lagged PD responses.

Findings in this study underlie the importance of muscular blockade monitoring among patients with severe aortic regurgitation during anesthetic induction.

**Registration:**

Name of the registry: Abnormal Cisatracurium Pharmacodynamics and Pharmacokinetics among Patients with Severe Aortic Regurgitation during Anesthetic Induction. Trial registration number: ChiCTR1800019654. Date of registration: November 20th 2018.

## Background

Cisatracurium besilate (NIBEX, 51 W89) is a nondepolarizing neuromuscular blocking agent widely used in anesthesia and ICU [1, 2]. About 77% of total clearance of cisatracurium undergoes Hoffmann degradation, which is characterized by spontaneous temperature- and pH-dependent chemical degradation in plasma and tissues [3]. Tetrahydropapaverine, the major metabolite of cisatracurium, has no neuromuscular blocking effect. In addition, histamine release reports are less seen in cistracurium than in other muscle relaxants [4].

Aortic regurgitation (AR) is a disorder in which the aortic valve fails to close properly and some blood flows back into the left ventricle during diastole [5]. According to the American Heart Association and the American College of Cardiology, severe aortic regurgitation is diagnosed in patients with ≥0.3cm^2^ regurgitant orifice area or ≥ 50% regurgitant fraction [6, 7]. The LV systolic function begins to aggravate if the disease developes naturally. Prognosis depends on the severity of regurgitation [6]. Surgery is proved to be the optimal treatment to protect left ventricle function and extend life expectancy for patients with severe AR [8, 9].

Compared with other muscle relaxants, cisatracurium is more favorable during anesthetic induction among patients with severe AR because of its low propensity to cause adverse cardiovascular effect [10]. Previous studies proved that the drug effect of cisatracurium would be compromised by some types of heart diseases with abnormal hemodynamics [11, 12]. The abnormal hemodynamics of aortic regurgitation, which is characterized by regurgitation into the left ventricle during diastole, may cause pharmacological change of cisatracurium in severe AR pateints. Thus it’s necessary to determine the pharmacodynamics (PD) and pharmacokinetics (PK) of cisatracurium in severe AR patients and this study was aimed to provide evidence to decide whether the common administration method should be modified in consideration of clinical efficacy and safety for the severe AR population.

## Methods

### Subjects

This clinical trial (No. GDREC2015297H) was approved by the Guangdong Provincial People’s Hospital Ethics Committee. The online registration number was ChiCTR1800019654. Patients were informed the objectives and risks of this trial before the operations. Written informed consents were obtained from all individual participants. From December 2018 to April 2019, 32 patients were enrolled in this study and divided into 2 groups: the AR group (*n* = 16) and the control group (n = 16). Patients in the AR group suffered from severe AR according to the diagnostic criteria published by AHA and ACC [5, 7]. And they were going to undergo elective aortic valve replacement. Patients in the control group suffered from breast fibroadenoma or thyroid adenoma, and their heart structure and function were proved to be intact by clinical examination, electrocardiography and echocardiography. And they underwent elective excision of breast fibroadenoma or thyroid adenoma.

Inclusion criteria for both groups are as follows:

1. age 20–60 years old;
2. BMI 20–24 kg/m^2^;
3. American Society of Anesthesiologists grade I or II.

Patients were excluded if they show any signs below:

1. history of allergy to cisatracurium or other anesthetics in this trial’s protocol;
2. compromised renal, liver or neuromuscular function.

### Preparation before anesthesia

Demographic information was collected and routine laboratory tests were applied in each case. Patients were prohibited from eating for 8 h and drinking for 4 h before elective surgeries. No preoperative anesthetic was administered before anesthesia.

### Anesthetic induction

Anesthetic induction was conducted intravenously in every subject through a peripheral catheter in the median cubital vein. 0.05 mg/kg midazolam, 1.5–2.5 mg/kg propofol and 5 μg/kg fentanyl were administered by bolus. After loss of eyelash reflex and unresponsiveness to oral instructions in the subject, a bolus of 0.15 mg/kg cisatracurium was injected. 4-6 mg/kg/h propofol and 0.05–0.1 μg/kg/min remifentanyl were infused to maintain stable hemodynamics and sufficient anesthesia.

### Neuromuscular monitoring

Pharmacodynamics was assessed by the Train-of-Four (TOF) tests. The negative and positive electrodes of TOF-Watch SX (Organon, Ireland) were placed on the ulnar side of the left forearm after skin cleaning. Calibration is carried out by single stimuli at 1 Hz upon the adductor pollicis muscles for 3 min in a row before the administration of cisatracurium and the start of TOF stimulation at 2 Hz every 15 s. TOF-Watch SX Monitor (version 2.2 int) was a software used to record the values of T_1_, T_2_, T_3_, T_4_ every 15 s. The values of T_4_, T_3_, T_2_ and T_1_ will decrease after muscle relaxants begin to take effect. T_4_, T_3_, T_2_ and T_1_ will become zero in turn as neuromuscular blockade increase. Finally T_1_ equals zero when skeletal muscles reach maximal blockade. Therefore, the onset time is defined as the interval between the moment cisatracurium is adminstered and the moment T_1_ first reaches zero.

### Blood sampling

Arterial blood samples (about 5 ml in each) were collected before (time 0) and at 1, 2, 4, 8, 12, 16, 20 min after cisatracurium administration. Blood samples were transferred to heparin-primed centrifuge tubes and centrifuged at 9000 *g* for 3 min (4 °C). Then the supernatant was mixed with 100 μl H_2_SO_4_ immediately and stored at − 80 °C until analysis.

Acetonitrile (800 μl) was used to deproteinize plasma samples (200 μl). The mixture was vortexed for 3 min and centrifuged at 15,000 *g* for 15 min. The resulting supernatant was obtained and dried using Eppendorf Concentrator Plus (Hamburg, Germany). The residue was reconstituted in a solution of water/acetonitrile [50:50(v/v);200 μl] before being centrifuged at 15,000 *g* for 15 min (4 °C). An aliquot of supernatant (5 μl) was injected into the UPLC-QTOF/MS system for quantification analysis.

### Quantification of plasma cisactracurium by UPLC-QTOF/MS system

An UPLC-QTOF/MS system equipped with an Acquity UPLC device and a Xevo G2 QTOF mass spectrometer (Waters, Milford, MA) was used to quantify the concentration of cisatracurum. A BEH column (2.1x50mm, 1.7 μm; Waters) was used to perform chromatographic separation. Formic acid (0.1%) in water (mobile phase A) and acetonitrile (mobile phase B) at a flow rate of 0.45 ml/min were used perform the gradient elution, which was 5% B at 0 to 1 min, 5 to 85% B at 1 to 3 min, 85% B at 3 to 3.5 min, and 85 to 5% B at 3.5 to 4 min. Cisatracurium concentration was quantified by full scan analysis and extracted ion chromatograms reported by MassLynx software (version 4.1; Waters).

### Limit of quantification and precision

The limit of quantification was defined as the final compound concentration with a signal-to-noise ratio larger than 10. Precision equals standard deviation of measured concentration divided by mean of measured concentration. A 1/× 2 weighted linear least-squares regression method was used to determine the linearity of the standard curve.

### PK analysis

**Fig. 1:**
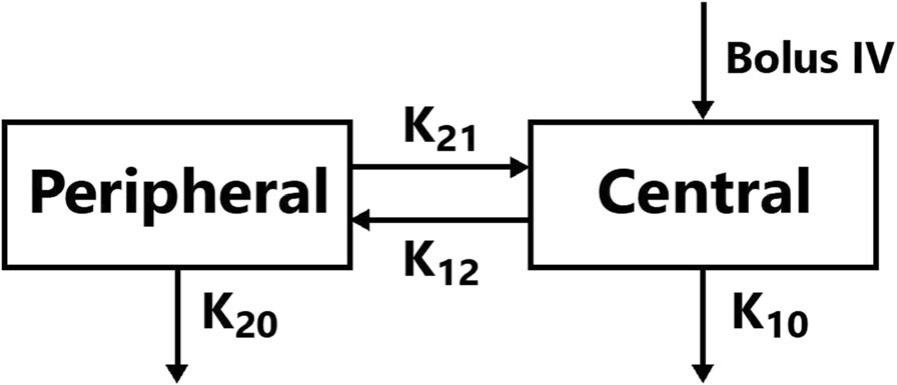
Schematic diagram of a conventional two-compartment model. IV, intravenous; K_12_, transfer rate constant from central compartment to peripheral compartment; K_21_, transfer rate constant from peripheral compartment to central compartment; K_10_, elimination rate constant for central compartment; K_20_, elimination rate constant for peripheral compartment

A conventional two-compartment model was constructed by WinNonlin software (Pharmsight, Mountain View, CA, USA) to analyze the PK data for cisatracurium (Fig. 1) [13].

### Statistical analysis

Categorical variables were represented as percentage and were tested by Pearson chi-squared analysis. Data of continuous variables were presented as mean ± standard deviation (SD). The unpaired Student’s t-test was performed to analyze the differences in PK and PD parameters between AR and control groups. The level of significance was set at *P* < 0.05. IBM SPSS Statistics 22.0 was used to conduct statistical calculation.

## Results

### Baseline data

Between the AR and control groups, there was no significant difference in demographic profile including age, gender, BMI and comorbid diseases (Table 1). In addition, no statistical difference was found between the two groups in parameters derived from blood gas analysis (Table 2).

**Table 1:** Demographic Profile

Parameter	Control (*n* = 16)	AR (*n* = 16)
Male/Female (n)	8/8	8/8
Age (years)	54.54 ± 9.07	52.88 ± 10.03
BMI (kg/m^2^)	21.74 ± 1.14	21.64 ± 1.22
Comorbid diseases	no	no

**Table 2:** Parameters Derived from Blood Gas Analysis

Parameter	Control (*n* = 16)	AR (*n* = 16)
Body Temperature (°C)	36.37 ± 0.10	36.35 ± 0.15
Blood pH	7.40 ± 0.03	7.39 ± 0.03
Hemoglobin (g/L)	137.88 ± 4.86	140.13 ± 6.54
Hematocrit (%)	40.17 ± 2.44	41.92 ± 3.28

### Abnormal cisatracurium PK in patients with severe AR

**Fig. 2:**
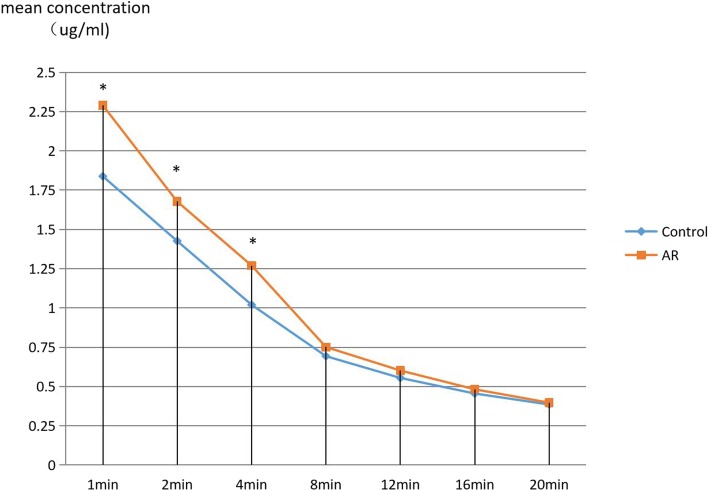
Mean plasma concentration vs time curves between the control and AR groupswere shown in this figure. The 1, 2, 4-min time points were marked with asteriks, indicating statistical differences in the mean plasma concentration between two groups (*P* < 0.05)

Figure 2 is a diagram presenting plasma concentration vs. time curves for both groups. Mean plasma cisatracurium concentration was significantly higher (*P* < 0.05) at early time points (1,2 and 4 min after cisatracurium injection) in the severe AR group than in the control group.

The mean values for the PK parameters derived from the conventional two-compartment model for both groups were presented in Table 3. A significant decrease in the intercompartmental transfer rate K_12_, K_21_ and T_1/2α_(*P* < 0.01) was seen in the AR group compared with the control group. Meanwhile, This finding is consistent with higher plasma concentrations of cisatracurium (at 1,2 and 4 min) in the AR group than in the control group. PK parameters evaluating drug elimination such as K_10_,K_20_ and T_1/2β_ were unaltered statistically in the AR group.

**Table 3:** Pharmacokinetic Parameters Derived from the Conventional Two-compartment Model after Administration of Cisatracurium

Parameter	Control	AR
K_10_ (1/min)	0.06 ± 0.01	0.07 ± 0.01
K_20_ (1/min)	0.04 ± 0.01	0.04 ± 0.01
K_12_ (1/min)	0.26 ± 0.02	0.19 ± 0.01*
K_21_ (1/min)	0.19 ± 0.01	0.11 ± 0.01*
V_1_ (ml/kg)	56.68 ± 7.79	52.34 ± 5.67
V_2_ (ml/kg)	41.31 ± 6.26	38.90 ± 5.48
T_1/2α_ (min)	1.52 ± 0.12	2.56 ± 0.16*
T_1/2β_ (min)	21.82 ± 3.02	23.76 ± 3.34
CL (ml/min/kg)	6.76 ± 1.36	7.06 ± 1.51
AUC (min*μg/ml)	23.81 ± 5.14	28.79 ± 6.25*

The mean values of PK parameters for both groups were shown here. All data were presented as means±SD. Statistcal differences between the two groups were indicated by asteriks (**P < 0.01). K_10_, elimination rate constant for central compartment; K_20_, elimination rate constant for peripheral compartment; K_12_, transfer rate constant from central compartment to peripheral compartment; K_21_, transfer rate constant from peripheral compartment to central compartment; V_1_, volume of the central compartment; V_2_, volume of the peripheral compartment; T_1/2α_, distribution half-life; T_1/2β_, elimination half-life; *CL* clearance; *AUC* area under the curve

### Abnormal cisatracurium PD in patients with severe AR

**Fig. 3:**
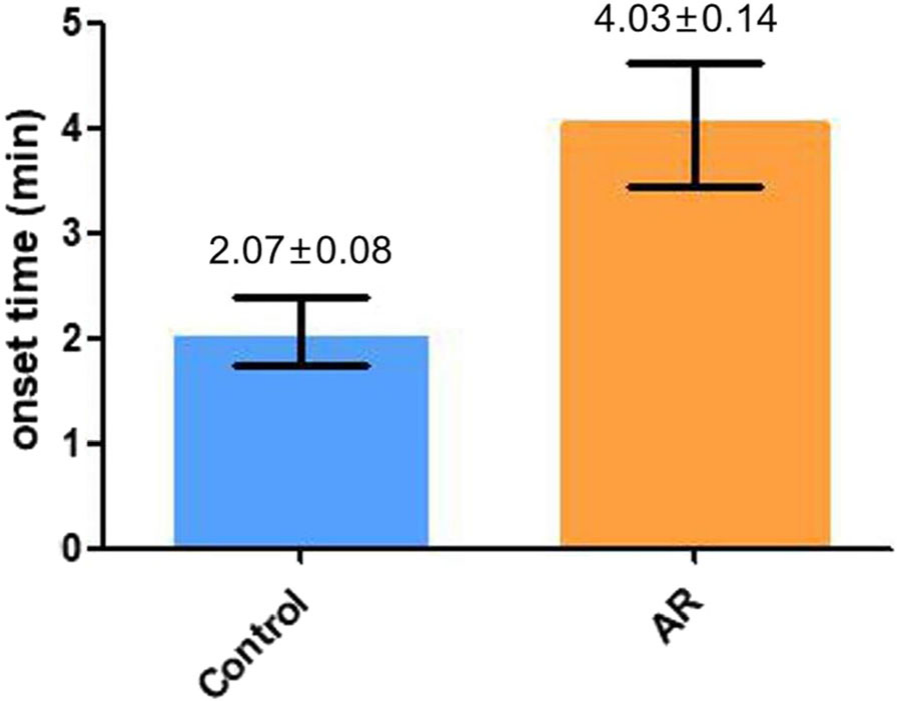
A comparison of onset time of the control and AR group

A column diagram depicting the onset time of two groups was shown in Fig. 3. The onset time of the control group and AR group were 2.07 ± 0.08 min and 4.03 ± 0.14 min respectively. The onset time of cisatracurium was significantly prolonged in severe AR patients compared with control patients (*P* < 0.05).

## Discussion

This is the first study examining the PK and PD of cisatracurium in patients with severe AR.

Cisatracurium mainly metabolizes by Hoffmann degradation, which is a spontaneous temperature- and pH-dependent chemical degradation in plasma and tissues [2, 3]. Thus, cisatracurium is barely affected by differences of liver and kidney function among patients so it helps us focus on the drug distribution difference between the two groups and make it easier to find the link between delayed drug distribution and lagged PD response.

The conventional two-compartment model was proved to precisely depict the PK profile of cisatracurium in previous studies [11, 12]. An elevation in plasma concentration of cisatracurium and slower transfer rate between the central and peripheral compartments were observed in the AR group. These PK characteristics are consistent with severe AR patients’ prolonged onset time (4.03 ± 0.14 min in AR group vs. 2.07 ± 0.08 min in control group). On the contrary, PK parameters evaluating drug elimination such as K_10_,K_20_ and T_1/2β_ were unaltered statistically in the AR group. This finding is consistent with the fact that cisatracurium metabolizes mainly by pH- and temperature-dependent Hoffmann reaction [4, 14] since no statistical difference was found in the blood gas analysis. Abnormal hemodynamics in severe AR patients could contribute to markedly altered distribution of cisatracurium but could not affect its elimination condition. Thus it’s reasonable to infer that the higher plasma concentration of cisatracurium resulted from impaired drug distribution instead of altered drug elimination [15]. Slower distribution of cisatracurium caused by severe AR’s abnormal hemodynamics underlies the lagged onset of maximal neuromuscular blockade.

The cause for the impaired transfer rate between the central and peripheral compartments in severe AR patients was not clearly elucidated before. But according to the typical abnormal pathology and pathophysiology of severe aortic regurgitation, it is reasonable to infer that the abnormal diastolic regurgitation primarily leads to reduction of forward blood flow and slower distribution of cisatracurium. And this study provided PD and PK evidence to suppor that viewpoint.

The sampling schedule (0–20 min) seems be short and could raise the doubt that if it’ll limit the scientific significance of this study. Collecting blood samples at eight time points during the 20-min sampling schedule proved sufficient to elaborate on the PK profile of cisatracurium due to two reasons. Firstly, the marked differences in plasma concentration of cisatracurium between the two groups were observed in the early 4 min after administration of cisatracurium. Secondly, the onset time to maximal neuromuscular blockade for both groups were both within the early 4 min though they were statistically different. Hence, it is assuring to say that the 20-min sampling schedule is long enough to examine the PK and PD characteristics in this trial.

A potential limitation of this study is that we do not have the post-operative PK and PD parameters of intravenous bolus of cisatracurium because it is against ethical code and clinical practice protocol to repeat the test after surgeries with the only purpose of collecting data. Our inferences of the impact of severe AR on PK and PD will be proved if pre-operative and post-opeartive parameters of PK and PD are found to be statistically different. Therefore, it’s worth to investigate in the future whether patients who have intact prosthetic aortic valves will present normal cisatracurium PK and PD characteristics when they undergo non-cardiac surgeries.

Out of clinical safety concern, anesthesiologists should be aware of the changes in PK and PD properties resulting from diseases and pathophysiological conditions. In clinical practices, it’s common for them to perform endotracheal entubation 2 minutes after the intravenous injection of 0.15 mg/kg cisatracurium without TOF monitoring [16]. Based on the findings in this study, 2 minutes are sufficient for patients with intact cardiac structure and function to obtain perfect neuromuscular blockade. However, severe AR patients require at least 4 min to obtain the same effect. Therefore, muscular blockade monitoring is essential for these patients during anesthetic induction.

## Conclusions

In conclusion, this study demonstrated that by PD and PK anaylsis severe AR impaired distribution of cisatracurium from central to peripheral compartment and caused lagged PD responses. These findings underlie the importance of neuromuscular blockade monitoring among patients with severe aortic regurgitation during anesthetic induction.

## Data Availability

The datasets during and analysed during the current study are available from the corresponding author on reasonable requests.

## Abbreviations

AR: Aortic Regurgitation
PD: Pharmacodynamics
PK: Pharmacokinetics
TOF: Train of Four
